# Computational screening of FDA-approved drugs to identify potential TgDHFR, TgPRS, and TgCDPK1 proteins inhibitors against *Toxoplasma gondii*

**DOI:** 10.1038/s41598-023-32388-9

**Published:** 2023-04-03

**Authors:** Zahra Gharibi, Behzad Shahbazi, Hamed Gouklani, Hoda Nassira, Zahra Rezaei, Khadijeh Ahmadi

**Affiliations:** 1 Infectious and Tropical Diseases Research Center, Hormozgan Health Institute, Hormozgan University of Medical Sciences, Bandar Abbas, Iran; 2 Molecular Medicine Department, Biotechnology Research Center, Pasteur Institute of Iran, Tehran, Iran; 3 Polymer Division, Department of Chemistry, Faculty of Science, University of Zanjan, Zanjan, Iran; 4 Professor Alborzi Clinical Microbiology Research Center, Shiraz University of Medical Sciences, Shiraz, Iran

**Keywords:** Computational biology and bioinformatics, Drug discovery

## Abstract

*Toxoplasma gondii* (*T. gondii*) is one of the most successful parasites in the world, because about a third of the world's population is seropositive for toxoplasmosis. Treatment regimens for toxoplasmosis have remained unchanged for the past 20 years, and no new drugs have been introduced to the market recently. This study, performed molecular docking to identify interactions of FDA-approved drugs with essential residues in the active site of proteins of T. *gondii* Dihydrofolate Reductase (TgDHFR), Prolyl-tRNA Synthetase (TgPRS), and Calcium-Dependent Protein Kinase 1 (TgCDPK1). Each protein was docked with 2100 FDA-approved drugs using AutoDock Vina. Also, the Pharmit software was used to generate pharmacophore models based on the TgDHFR complexed with TRC-2533, TgPRS in complex with halofuginone, and TgCDPK1 in complex with a bumped kinase inhibitor, RM-1–132. Molecular dynamics (MD) simulation was also performed for 100 ns to verify the stability of interaction in drug–protein complexes. Molecular Mechanics Poisson-Boltzmann Surface Area (MMPBSA) analysis evaluated the binding energy of selected complexes. Ezetimibe, Raloxifene, Sulfasalazine, Triamterene, and Zafirlukast drugs against the TgDHFR protein, Cromolyn, Cefexim, and Lactulose drugs against the TgPRS protein, and Pentaprazole, Betamethasone, and Bromocriptine drugs against TgCDPK1 protein showed the best results. These drugs had the lowest energy-based docking scores and also stable interactions based on MD analyses with TgDHFR, TgPRS, and TgCDPK1 drug targets that can be introduced as possible drugs for laboratory investigations to treat *T. gondii* parasite infection.

## Introduction

*Toxoplasma gondii* is an obligate intracellular protozoan parasite with a polar apical complex and an attachment to the host cell membrane^1,2^. *Toxoplasma gondii* infects almost all mammals and birds, including humans, domestic animals, and wild animals. In some infected people, mild symptoms like an influenza-like illness, such as muscle aches and swollen lymph nodes, appear for weeks or months^3^. Eye problems are also seen in a few patients. Some patients with weakened immune systems may develop neurological problems such as imbalance and coordination of muscles and organs^1,4^. The acute form of the disease may be associated with fever and lymphocytosis. In the rare acute form of the disease, symptoms of pneumonia, general muscle involvement, and even death occur^5^. Cats are the definitive host for *T. gondii*. Humans and other mammals act as intermediate hosts. Drugs used to treat toxoplasmosis have toxic side effects and need long periods of time, ranging from weeks to more than a year. The need for long-term treatment and the risk of recurrence of the disease is partly due to inefficiency against *T. gondii* tissue cysts. Challenges to creating a more effective treatment for toxoplasmosis include reducing toxicity, achieving therapeutic concentrations on the brain and eyes, shortening the duration, removing tissue cysts from the host, safety in pregnancy, and developing a formula that is available in inexpensive and practical sources^6^. Drug therapies affect the reproductive stage of tachyzoite by inhibiting folic acid biosynthesis and protein synthesis. For decades, drug regimens for human toxoplasmosis have included sulfadiazine, pyrimethamine, and autocon, which are effective against other parasites, such as Plasmodium. Other drugs include spiramycin, azithromycin, and clindamycin. Trimethoprim and sulfamethoxazole both effectively combat the parasite, so cotrimoxazole, a combination of the two, is the preferred treatment of 3–4 weeks and is usually prescribed with folic acid to reduce the drug's side effects. Failure to treat existing medications may be due to long-term use. However, due to the complex life cycle of the parasite, the development of resistance, and treatment failure in *Toxoplasma* chorioretinitis, encephalitis, and congenital infections is increasing and needs further investigation. The duration of initial treatment for Toxoplasmic encephalitis is at least six weeks, followed by secondary suppression until adequate recovery of the immune system. Long-term treatment is partly because current clinical drugs cannot eliminate the cystic stage of *T. gondii*. Drug development and administration are essential with a reliable understanding of the mechanism of action, the specific site of the target, and the consequences of its behavior on the host and the parasite^7^. Currently, approved drugs cannot clear chronic infections in the human body, so approximately one-third of the world's human population is at risk of reactivation, with potentially severe consequences. Drugs that have been successfully repurposed in research, along with the ever-increasing costs and failures of traditional drug discovery, have led to the emergence of a new field of drug repurposing^8^. Nowadays, scientists are trying to replace drugs with fewer and milder side effects with old drugs that do not respond to the treatment of some diseases or have high side effects^9^.

Studies suggest that calcium signaling pathways are important in *T. gondii* parasite, as calcium ionophores can stimulate microneme secretion and suppress some activities^10,11^. Calcium-dependent protein kinase 1 (CDPKs) is a family of serine/threonine kinases. Replication of *T. gondii* depends on its ability to invade host cells, which is partially mediated by CDPK1. TgCDPK1 is an essential regulator of calcium-dependent exocytosis in this opportunistic human pathogen. Suppression of this protein has been shown to control the calcium-dependent secretion of organelles called micronemes, thereby blocking essential phenotypes such as parasite motility, host cell invasion, and egress^12–14^. In some studies, TgCDPK1 is a potential new target in *T. gondii* for treating this infection^14–16^. Recent studies also have targeted parasite enzymes that are involved in protein synthesis. These enzymes play an essential role in proteostasis^17–19^. Prolyl-tRNA synthetases (PRS) are among the enzymes that have received much attention. These enzymes charge tRNA molecules with L-pro for protein translation. In recent studies, parasite-encoded PRSs have been extensively evaluated using febrifugein and its derivatives, such as halofuginone^17,20^. Apicomplexan parasites use the combined enzyme thymidylate synthase-Dihydrofolate Reductase (TS-DHFR) for folate metabolism and pyrimidine biosynthesis, which are essential for cell growth and proliferation. TS-DHFR is an attractive target for pharmacological interventions. Drugs that inhibit this drug target with their competitive inhibitory properties are widely used in the clinic to treat parasitic infections, including toxoplasmosis^21–23^. In this study, we have developed a computational approach to analyze the inhibitory effect of some promising drugs against *T. gondii* CDPK1, PRS, and DHFR proteins using molecular docking evaluation and MD simulation, where we can select useful drugs from all the investigated drugs. For this aim, we examined the interaction of 2100 FDA-approved drugs with three *T. gondii* CDPK1, PRS, and DHFR proteins individually. Finally, we identified the most important drugs with inhibitory effects on different proteins of *T. gondii*.

## Results

### Analysis of interactions of FDA-approved drugs with TgDHFR protein

**Figure 1:**
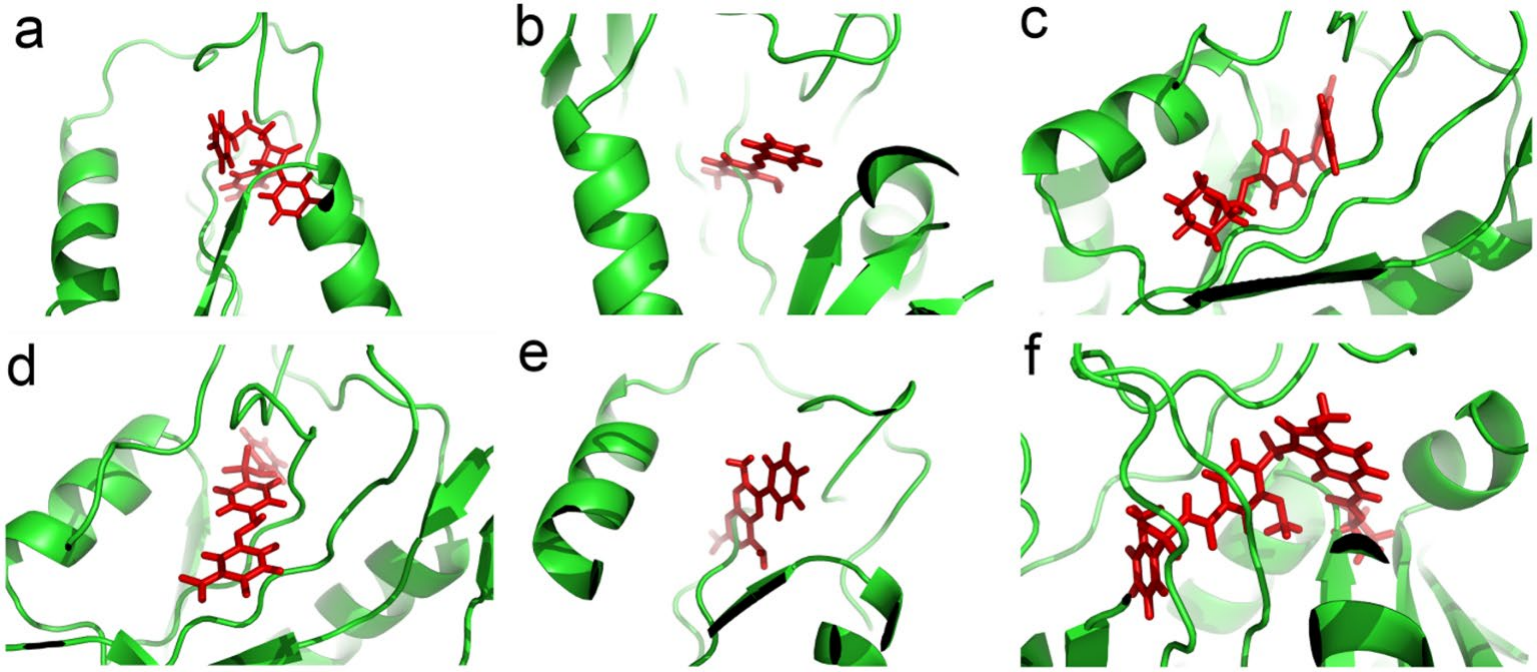
The best drugs with high affinity bind in the groove of the TgDHFR. (**a**) Ezetimibe, (**b**) Phenazopyridine, (**c**) Raloxifene, (**d**) Sulfasalazine, (**e**) Triamterene, (**f**) Zafirlukast. The figures are created by using PyMOL Version 1.1.

The binding energies of the selected FDA-approved drugs with the *T. gondii* DHFR (PDB ID: 6AOH) were investigated. Evaluation analysis of molecular docking results were scored based on the formation of H-bonds, the affinity scores, the interaction energies, and interaction with the amino acid residues present in the binding site of TgDHFR protein. We analyzed the docking scores predicted by AutoDock Vina and ranked the top compounds in the first level of docking results screening. Compounds with low affinity scores were first rejected and not further analyzed. Drugs with high side effects such as anti-cancer drugs as well as expensive drugs were excluded from our drug list. Then, thoroughly analyzed and presented the interactions between the best compounds based on the docking results and the residues inside the protein binding pocket in Table 1. Among all compounds, Zafirlukast showed the highest binding affinity to the binding site of the protein (affinity score − 10.9037 kcal/mol). Ezetimibe, Raloxifene, Sulfasalazine, Triamterene, and Zafirlukast drugs showed lower binding energy compared to TRC-2533 (Table 1), which is an inhibitor of DHFR receptor (https://www.rcsb.org/structure/6AOH). The 3D structural views of the ligand–protein interactions are provided in Fig. 1. Evaluating the interactions of different ligands in the active site of the TgDHFR protein based on docking results showed that the Val9, Ala10, Met11, Tyr157, and Ile17 residues play a crucial role in the placement of these drugs in the binding site of DHFR protein. We took the top ranked compounds for further evaluation to MD simulations.

**Table 1:** Summary of the best docking interactions of the FDA-approved drugs against the TgDHFR protein. Interactions were analyzed and visualized using LigPlot + v.1.4.5.

Compound	Docking interaction	Binding Energy (kcal/mol)	Interpretation
TRC-2533		− 8.2	Hydrogen bond: Asp31, Thr172, Val8, Val151 Hydrophobic bond: Pro88, Leu23, Val9, Ala10, Phe35, Met87Phe32
Ezetimibe		− 10.1818	Hydrogen bond: Val9, Met11, Ile17 Hydrophobic bond: Ala10, Gly18, Ile171, Val151, Leu23, Trp25, Val182, Phe183, Ala154, Glu158, Pro185, Thr83, Gly153, Phe184
Phenazopyridine		− 7.60308	Hydrogen bond:Ala10, Tyr157 Hydrophobic bond: Val8, Leu23, Met11, Ile171, Thr172
Raloxifene		− 10.8928	Hydrogen bond: Val8, Val9, Ala10, Ile17, Tyr157, Leu169 Hydrophobic bond: Met11, Ile171, Val151, Val150, Leu161, Ala166, His168, Cys6, Thr83
Sulfasalazine		− 10.1582	Hydrogen bond: Val9, Ala10, Met11, Ile17, Leu248 Hydrophobic bond: Thr83, Ser86, Tyr157, Thr172, Val246, Leu169, Arg173, Ile192, Phe187, Phe184
Triamterene		− 8.74162	Hydrogen bond: Ala10, Tyr157, Thr172 Hydrophobic bond: Val8, Met11, Trp25, Leu23, Ile171
Zafirlukast		− 10.9037	Hydrogen bond: Val9, Ala10, Met11, Tyr157, Gly153, Gly80, Leu23, Ile17 Hydrophobic bond: Pro24, Phe184, Leu193, Phe187, Leu248, Tyr170, Leu169, Val8, Thr172, Val151, Leu156, Met79, Val78, Thr83, Gly18, Trp25

### Binding interactions of FDA-approved drugs with TgPRS protein

**Figure 2:**
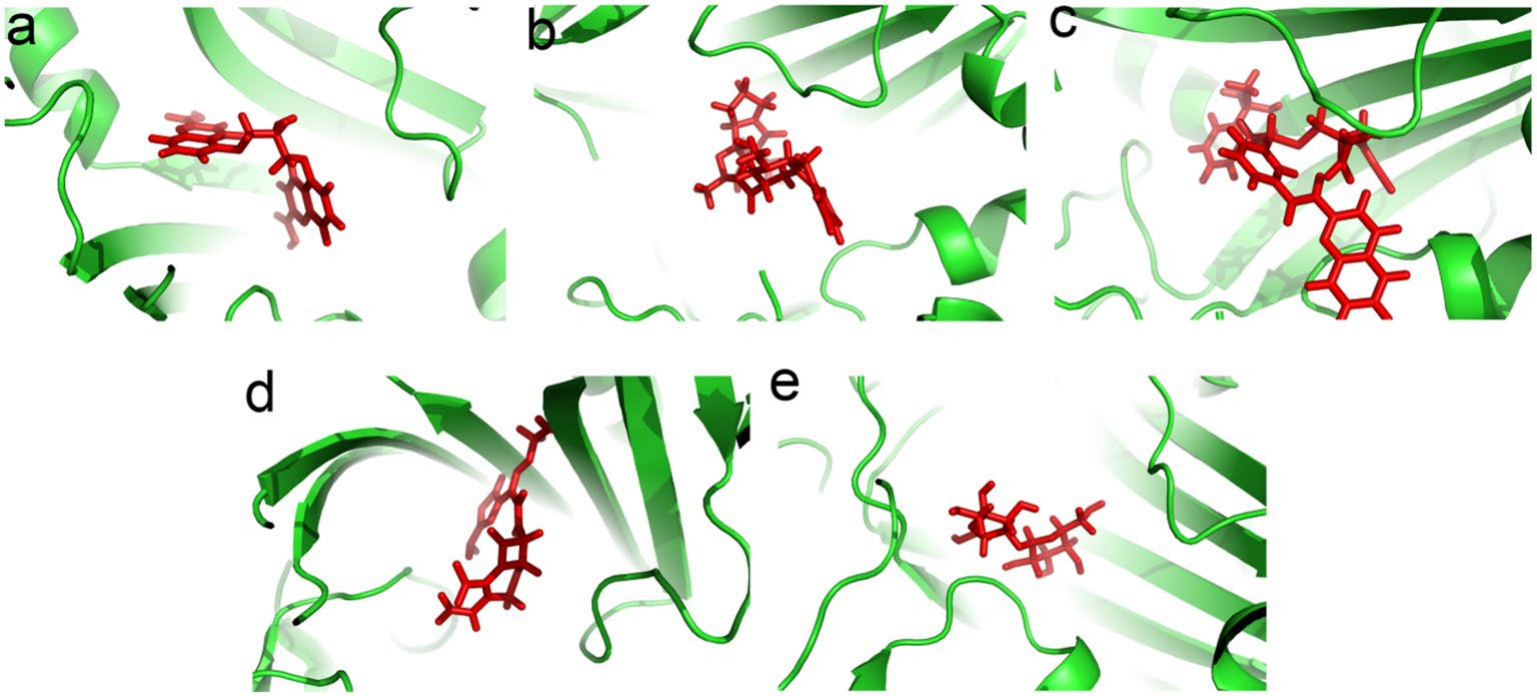
The best drugs with high affinity bind in the groove of the TgPRS protein. (**a**) Cromolyn, (**b**) Ergotamine, (**c**) Montelukast, (**d**) Cefexim, (**e**) Lactulose. The figures are created by using PyMOL Version 1.1.

Docking analysis was done between 2100 FDA-approved drugs with TgPRS protein. The strongest interactions between the selected drugs and TgPRS (PDB ID = 5XIQ) are provided in Table 2. Cromolyn, Ergotamine, Montelukast, Cefexim, and Lactulose bound efficiently to the TgPRS protein. Cromolyn, Ergotamine, and Montelukast drugs showed lower binding energy (≤ Binding Energy: − 10.3 kcal/mol) compared to Halofuginone (Binding Energy =  − 10.2 kcal/mol), which is an inhibitor of TgPRS receptor (https://www.rcsb.org/structure/5XIQ) (Table 2). Montelukast showed the lowest binding energy and, thus the highest affinity to bind to the active site of the TgPRS protein (Binding Energy: − 11.0533 kcal/mol). Cromolyn, Ergotamine, and Montelukast were selected based on the results of AutoDock Vina as the strongest interaction (Lowest binding energy) with the TgPRS protein. It also identified the drugs Cefexim and Lactulose using the Pharmit server based on pharmacophore calculations as effective drugs against TgPRS protein with a structure similar to halofuginone compound that is TgPRS inhibitor. Our other criteria for the selection of drugs were low side effects and cost-effectiveness. For example, anticancer drugs that have many side effects were removed from our drug list, as well as expensive drugs. Evaluation of the interactions of different ligands in the active site of the TgPRS protein based on docking results showed that the Thr439, Arg470, His491, His560, Cys591, Gly590, Ala556, Ala557, and Trp487 residues present at the predicted active site of the TgPRS protein, and have a key role in the interaction of ligand–protein. 3D structural visualizations of the drug–protein site interactions are shown in Fig. 2.

**Table 2:** Summary of the best docking interactions (Binding Energy:** ˂** − 10.0 kcal/mol) of the FDA-approved drugs against the TgPRS protein. Interactions were analyzed and visualized using LigPlot + v.1.4.5.

Compound	Docking interaction	RMSD score	Binding Energy (kcal/mol)	Interpretation
Halofuginone			− 10.2	Hydrogen bond: Arg470, Thr439, His560, Glu441 Hyrohpobic bond: Phe415, Glu418, Pro438, Glu489, Trp487, Trp589, His491, Gly590, Thr558, Phe534
Cromolyn			− 10.3126	Hydrogen bond: Thr439, Arg470, Ala556, Thr592 Hyrohpobic bond: Arg481, Phe485, His560, Phe534, Gln441, His491, Trp487, Glu489, Thr558, Trp589, Ser588, Cys591, Gly590, Ala557, Arg594
Ergotamine			− 10.5939	Hydrogen bond: His560, Arg470 Hyrohpobic bond: Arg481, Thr439, Phe415, Phe534, Gln532, Gln555, Glu489, Thr558, Cys591, Gly590, Thr592
Montelukast			− 11.0533	Hydrogen bond: His491, Thr439 Hyrohpobic bond: Leu405, Phe415, Glu441, Arg470, Trp487, Glu489, Phe485, Phe534, His560, Ser588, Trp589, Gly590, Thr558, Ala556, Cys591, Ala557, Gln555, Thr592, Arg594
Cefexim		0.508391142		Hydrogen bond: Arg470, Thr439, Glu441, Gly590, Ala557 Hyrohpobic bond: Glu489, His491, Trp487, Trp589, Ala556, Tyr511, Ser559, His560, Phe534
Lactulose		0.392093897		Hydrogen bond: Glu418, Pro438, Arg470, Trp487, Thr439, His560 Hyrohpobic bond: Val469, Phe485, Val468, Glu489, Thr558

### Analysis of interactions of selected drugs with TgCDPK1

**Figure 3:**
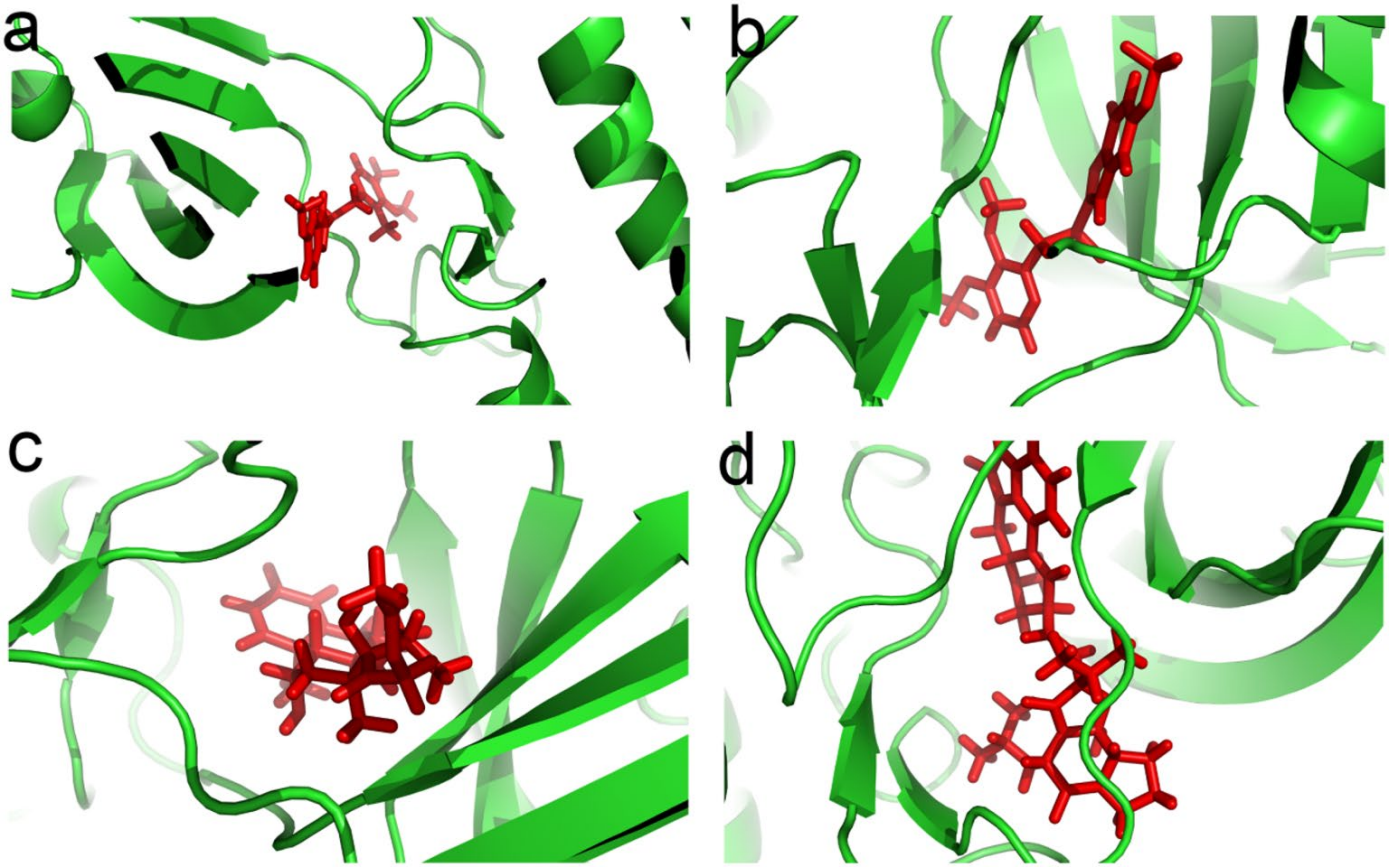
The best drugs with high affinity bind in the groove of the TgCDPK1. (**a**) Omeprazole, (**b**) Pantoprazole, (**c**) Betamethasone, (**d**) Bromocriptine. The figures are created by using PyMOL Version 1.1.

The binding energies of the selected FDA-approved drugs with the TgCDPK1 protein (PDB ID: 3SX9) were studied. The results of drug interactions with the protein were ranked based on affinity scores, interaction energies, number of bonds, and interaction of ligands with amino acid residues in the active site of the protein. In the first screening stage, we relied on the docking scores predicted by AutoDock Vina and ranked the top compounds. Drugs with lower affinity and more positive energy were first rejected and not further analyzed. The results of the interactions between the drugs and the residues inside the active site and the RMSD score related to the virtual screening of the Pharmit server, were thoroughly analyzed and presented in Table 3. Among all drugs, Pantoprazole showed the highest binding affinity to the binding site of the protein (affinity score − 6.03039 kcal/mol) which was close to the binding affinity of RM-1–132 (affinity score − 6.1 kcal/mol) as an inhibitor of TgCDPK1 receptor in the study of Larson et al.^24^. Betamethasone and Bromocriptin, as effective drugs against TgCDPK1 protein (with a structure similar to RM-1–132 compound) were also detected by the Pharmit server based on pharmacophore calculations (Table 3). The 3D structural views of the protein–ligand interactions are presented in Fig. 3. Evaluating the interactions of different ligands in the active site of the TgCDPK1 protein based on docking results showed that the Met112, Leu114, Gly128, Glu129, Val130, and Leu181 residues play a key role in the placement of these compounds in the active site. We took the top ranked drugs for further evaluation of MD simulations.

**Table 3:** Summary of the best docking interactions of the FDA-approved drugs against the TgCDPK1 protein. Interactions were analyzed and visualized using LigPlot + v.1.4.5.

Compound	Docking interaction	RMSD score	Binding Energy (kcal/mol)	Interpretation
RM-1–132			− 6.1	Hydrogen bond: Glu129, Tyr131, Glu135 Hydrophobic bond: Leu198, Leu114, Leu126, Asp195, Ile194, Lys80, Met112, Glu178, Val65, Leu57, Leu181, Ala78
Omeprazole			− 5.96554	Hydrogen bond: Glu129, Val130, Glu183 Hydrophobic bond: Ala78, Lys80, Val65, Leu181, Leu182, Arg192
Pantoprazole			− 6.03039	Hydrogen bond: Ala78, Met112 Hydrophobic bond: Leu114, Leu198, Leu126, Lys80, Ile194, Val65, Leu181, Leu57, Tyr131, Val130
Betamethasone		0.299921304		Hydrogen bond: Met112, Glu129, Leu181 Hydrophobic bond: Leu198, Leu114, Leu126, Val79, Lys80, Gly128, Ala78, Val65, Ile194
Bromocriptine		0.729647875		Hydrogen bond: Met112, Leu114, Gly128, Glu129, Val130, Gly133, Gly134, Tyr131, Hydrophobic bond: Tyr115, Val127, Lys113, Ala78, Thr132, Leu182, Leu180, Ile194,

### MD simulation of top scored complexes

MD is a computer simulation technique that investigates the physical motions of atoms and molecules. Evaluating the stability of the docked complexes Root Mean Square Fluctuation (RMSF), Root-mean-square Distance (RMSD), hydrogen bonds (H-bonds), radius of gyration (RG), and solvent-accessible surface area (SASA) were calculated. In MD simulation, atoms and molecules are allowed to interact for a fixed period. It provide a view of the dynamic evolution of the system and visualize the effect of ligand binding on protein structural changes. The highest estimated binding energy and the best orientation of the ligand in the active site were selected to perform MD simulations. In the interaction with the DHFR protein, drugs of Ezetimibe, Phenazopyridine, Raloxifene, Sulfasalazine, Triamterene, Zafirlukast, and DHFR-TRC-2533 complex were chosen for MD simulation.

RM-1–132-CDPK1, Omeprazole-CDPK1, Pantoprazole-CDPK1, Betamethasone-CDPK1, Bromocriptine-CDPK1 complexes were chosen to check the stability of drugs in the interaction with the CDPK1 protein. Halofuginone-PRS, Cromolyn-PRS, Ergotamine-PRS, Montelukast-PRS, Cefexim-PRS, and Lactulose-PRS complexes were also evaluated to analyze the stability of complexes.

**Figure 4:**
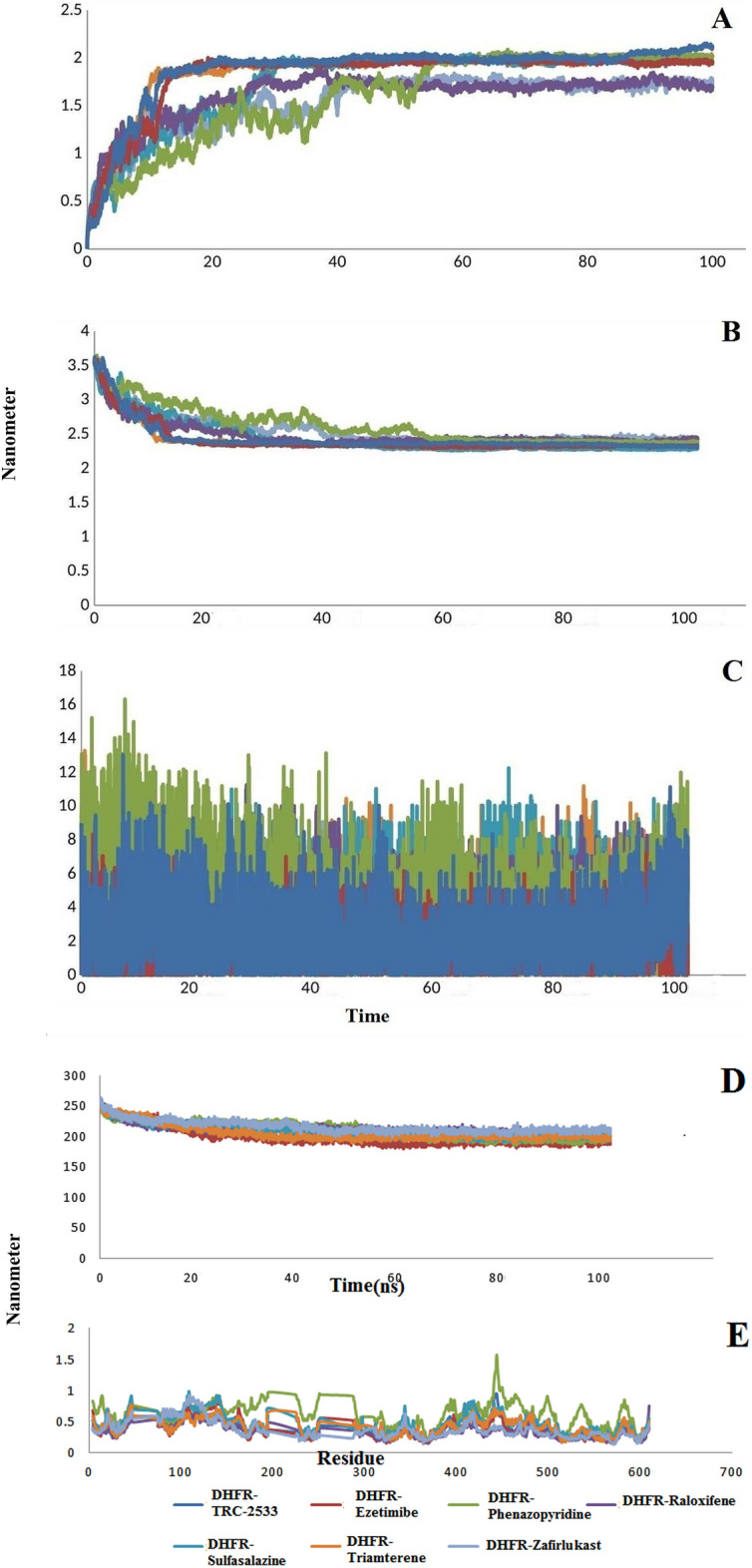
(**A**) RMSD values of DHFR docked complexes. (**B**) Rg values of DHFR docked complexes. (**C**) Number of H-bonds of DHFR docked complexes. (**D**) SASA values of DHFR docked complexes. (**E**) The RMSF values of DHFR protein in the simulated complexes compared to DHFR-TRC-2533 complex as control.

The RMSD results for drugs interacting with the TgDHFR protein showed that all drugs eventually reached stability (Fig. 4A). The average of this stability for the DHFR-TRC-2533 complex as control was 1.87 nm, and for drugs of Ezetimibe, Phenazopyridine, Raloxifene, Sulfasalazine, Triamterene, and Zafirlukast were 1.82, 1.60, 1.61, 1.74, 1.84, and 1.54 nm respectively. These results show that the most stable complexes are DHFR-Zafirlukast, DHFR-Phenazopyridine, and DHFR-Raloxifene. The Rg value evaluated the folding and compactness of the docked complexes. The analysis of the compactness of the docked complexes of drug-DHFR showed that all the complexes finally reached stability after about 40 ns and were not changed dramatically, the protein and ligands did not separate after this period and kept their connection which is a good indication of complexes' stability (Fig. 4B). The stability of the hydrogen bonds is critical in the interaction between the drug and the protein. The analysis of this parameter for drug-DHFR complexes showed that the hydrogen bonds for all complexes have low fluctuations and are stable during the simulation (Fig. 4C). Checking the exposed areas to the solution by SASA analysis confirmed the stability of the complexes. As shown in Fig. 4D, all the complexes are stable during the simulation. In addition, to analyze the fluctuations of the backbone atoms of structures of the complexes, we decided to perform RMSF analysis. In this analysis, the average value of fluctuation of each residue during the simulation is plotted. As shown in Fig. 4E, the RMSF values of all complexes were stable and indicated low fluctuation in most residues. The lowest fluctuations were related to the DHFR-Zafirlukast complex.

**Figure 5:**
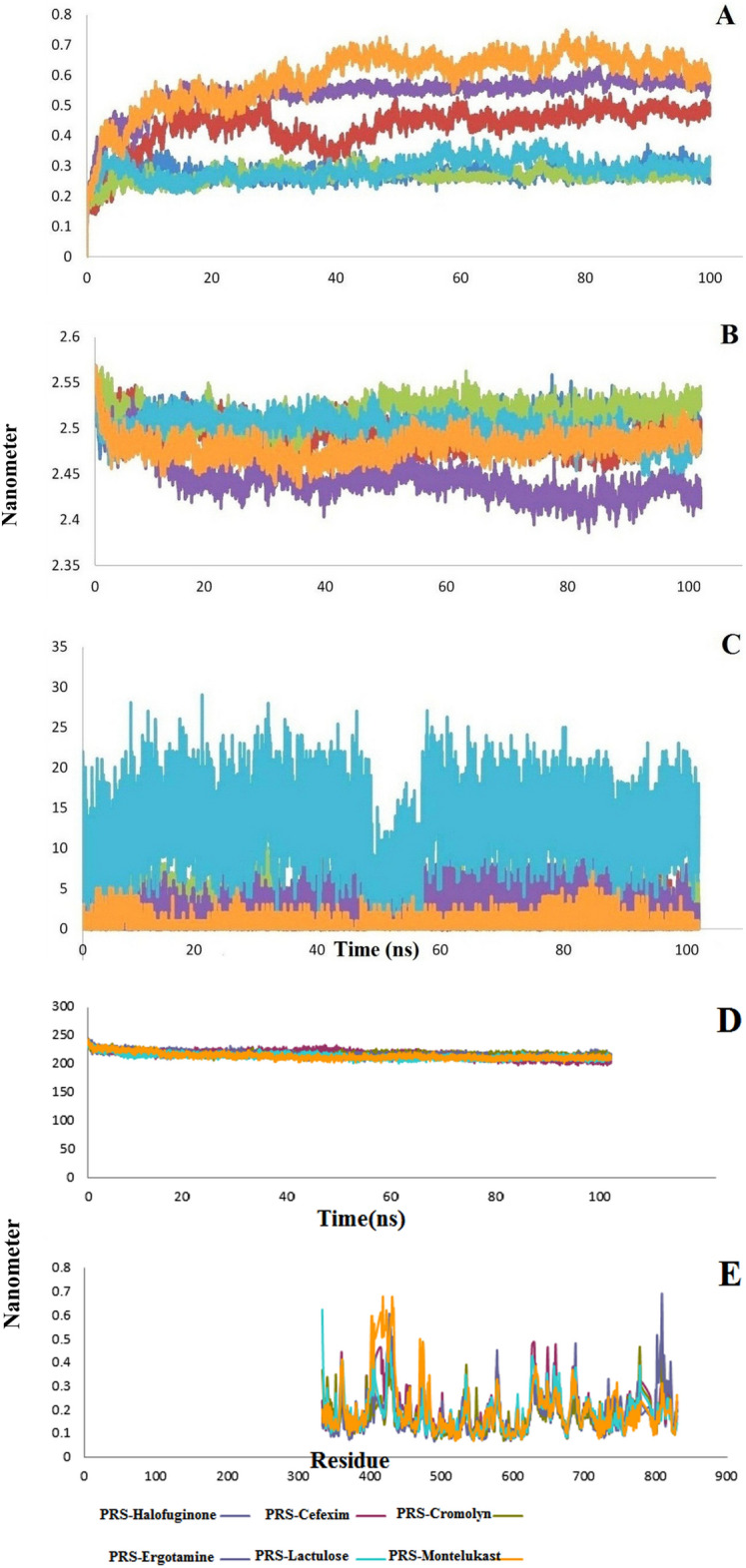
(**A**) RMSD values of PRS docked complexes. (**B**) Rg values of PRS docked complexes. (**C**) Number of H-bonds of PRS docked complexes. (**D**) SASA values of PRS docked complexes. (**E**) The RMSF values of PRS protein in the simulated complexes compared to PRS-Halofuginone complex as a control.

Molecular dynamics simulation was performed for PRS-Halofuginone, Cromolyn-PRS, Ergotamine-PRS, Montelukast-PRS, Cefexim-PRS, and Lactulose-PRS complexes up to 100 ns. The RMSD results showed that all the complexes were stable, with low fluctuations during the simulation (Fig. 5A). Halofuginone-PRS complex with RMSD average of.27 nm had good stability as control. Cromolyn and lactulose complexes are among the most stable complexes, with an average of 0.26 and 0.28 nm, respectively. Analysis of the compactness of the complexes during the simulation showed that the fluctuations are insignificant and the complexes are stable (Fig. 5B). The hydrogen bond stability was also checked during the simulation. The results showed that the complexes have stability in number of hydrogen bonds (Fig. 5C). The highest average number of hydrogen bonds is related to the Lactulose-PRS complex with 12.8 hydrogen bonds compared to the average number of bonds related to the Halofuginone-PRS complex as controlled with.9 hydrogen bonds. SASA is used to determining the surface accessible by solvent molecules. The average SASA values of the complexes were investigated during 100 ns MD simulation and were found to be 218.5, 217.2, 217.8, 216.5, 214.1, and 214.4 nm^2^, respectively.

The average SASA value of our selected complexes based on molecular docking and Halofuginone-PRS complex as the control was close , and all complexes were stable during the simulation (Fig. 5D). In addition, to analyze the fluctuations of the backbone atoms of the structures of the selected complexes, we decided to perform RMSF analysis. In this analysis, the average fluctuation value of each residue during the simulation was plotted (Fig. 5E). As shown in Fig. 5E, the RMSF values show minor fluctuations for most residues in the complexes.

**Figure 6:**
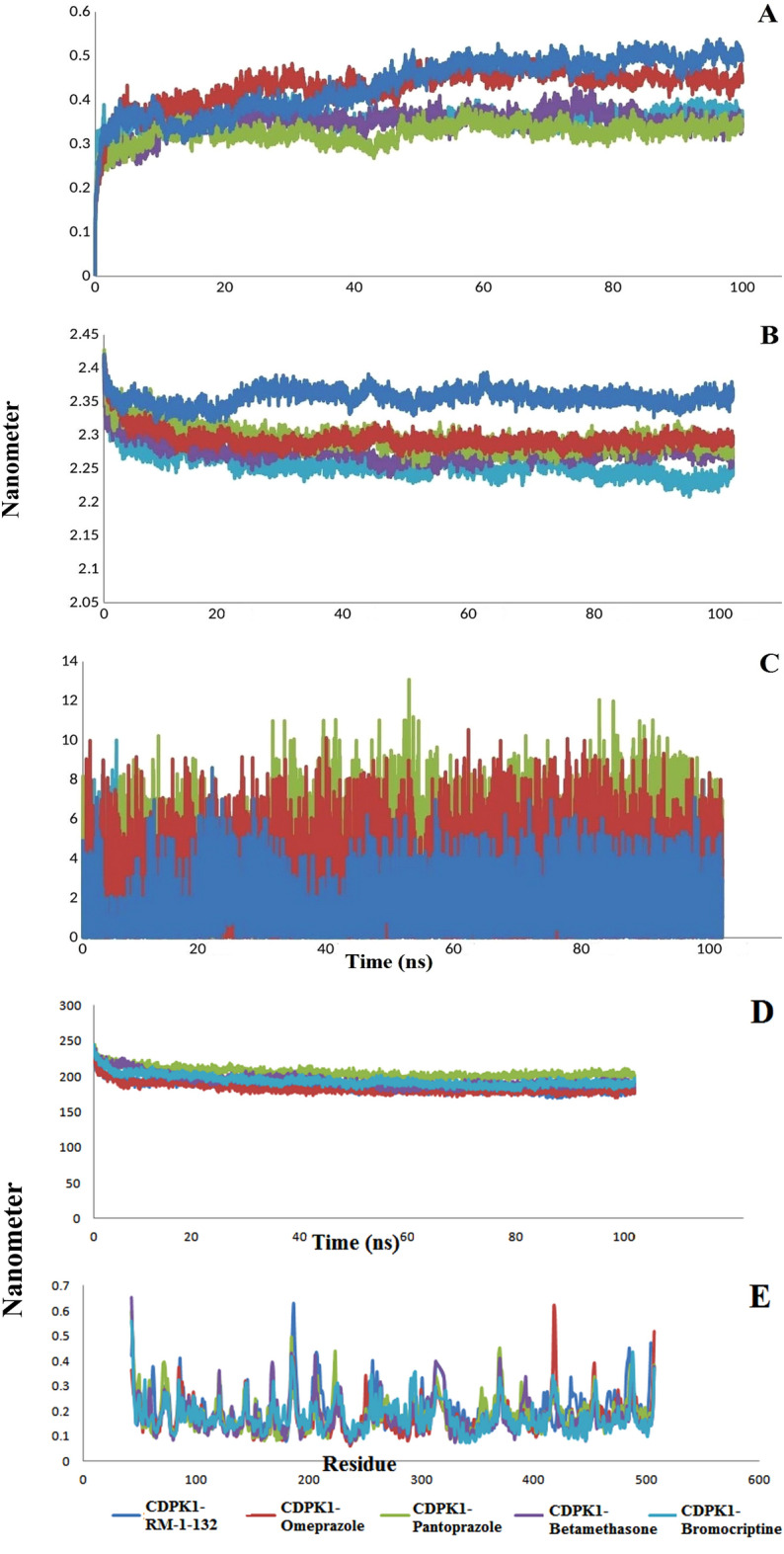
(**A**) RMSD values of CDPK1 docked complexes. (**B**) Rg values of CDPK1 docked complexes. (**C**) Number of H-bonds of CDPK1 docked complexes. (**D**) SASA values of CDPK1 docked complexes. (**E**) The RMSF values of CDPK1 protein in the simulated complexes compared to CDPK1-RM-1–132 complex as control.

Examination of the RMSD values showed that CDPK1-RM-1–132, Omeprazole-CDPK1, Pantoprazole-CDPK1, Betamethasone-CDPK1, Bromocriptine-CDPK1 complexes were stable during the simulation 100 ns. The average RMSD values were 0.43, 0.42, 0.32, 0.34, and 0.35 nm for CDPK1-RM-1–132, Omeprazole-CDPK1, Pantoprazole-CDPK1, Betamethasone-CDPK1, Bromocriptine-CDPK1, respectively (Fig. 6A). Also, the evaluation of the compactness of the complexes showed that are stable with an average Rg of 2.35, 2.29, 2.29, 2.27, and 2.25 nm (Fig. 6B). The docked complexes and their stability degrees were further evaluated by examining number of total hydrogen bonds during the simulation (Fig. 6C). The formation of hydrogen bonds and number of fundamental changes in the simulated complexes can be used to define the rigidity of the complexes. Average number of hydrogen bonds for CDPK1-RM-1–132, Omeprazole-CDPK1, Pantoprazole-CDPK1, Betamethasone-CDPK1, Bromocriptine-CDPK1 complexes were calculated as 1.65, 2.7, 4.2, 0.95, and 2.5, respectively. SASA parameter calculates the level of ligand–protein complexes that are directly accessible to solvent molecules. The average values of SASA for the docked complexes of CDPK1-RM-1–132, Omeprazole-CDPK1, Pantoprazole-CDPK1, Betamethasone-CDPK1, Bromocriptine-CDPK1 are 185.9, 183.2, 205.3, 194.1, and 194.1 nm^2^ respectively (Fig. 6D). Negligible fluctuations in values means that the docked complex is very tight and stable. In addition, complexes flexibility among amino acid residues were analyzed by evaluating the RMSF profile. The RMSF profiles of the docked complexes showed that the average RMSF of the complexes with minor fluctuations was below 0.2 nm. This result, as shown in Fig. 6E, is that all complexes are stable.

### MMPBSA binding-free energy of the simulated complexes

The binding energies were computed using the MMPBSA tool to analyze the molecular interactions of the DHFR-TRC-2533, DHFR-Ezetimibe, DHFR-Phenazopyridine, DHFR-Raloxifene, DHFR-Sulfasalazine, DHFR-Triamterene, and DHFR-Zafirlukast complexes for DHFR protein, PRS-Halofuginone, Cromolyn-PRS, Ergotamine-PRS, Montelukast-PRS, Cefexim-PRS, Lactulose-PRS complexes for PRS protein and CDPK1-RM-1–132, Omeprazole-CDPK1, Pantoprazole -CDPK1, Betamethasone-CDPK1, Bromocriptine-CDPK1 complexes for CDPK1 protein. The Van der Waals, electrostatic, polar solvation, SASA, streptavidin (SAV), and binding energies of complexes were calculated by the MMPBSA method. SASA of the complexes was calculated to predict the extent of conformational change during the interaction. As shown in Table 4, interactions between the drug-DHFR complexes were strong. The MMPBSA binding energy calculations exhibited high binding energies, especially for the Ezetimibe-DHFR (− 221.554 ± 55.106 kJ/Mol), Raloxifene-DHFR (− 272.645 ± 69.17 kJ/Mol), Sulfasalazine-DHFR (− 266.270 ± 12.67 kJ/Mol), Triamterene-DHFR (− 138.731 ± 94.791 kJ/Mol), and Zafirlukast-DHFR (− 270.513 ± 16.938 kJ/Mol) complexes. As shown in Table 4, the binding energy for these complexes are much more negative than the energy of the TRC-2533-DHFR complex (− 33.192 ± 16.517 kJ/Mol) as a control, which indicates a much stronger binding of drugs Ezetimibe, Raloxifene, Sulfasalazine, Triamterene, and Zafirlukast to the DHFR protein. Calculating the binding energy of Cromolyn-PRS, Ergotamine-PRS, Montelukast-PRS, Cefexim-PRS, and Lactulose-PRS complexes showed that all the selected drugs have strong binding to the PRS protein (Table 5). Among the complexes, Cromolyn-PRS, Cefexim-PRS, and Lactulose-PRS have a stronger bond to the PRS protein. Also, the MMPBSA analysis for the CDPK1-RM-1–132, Omeprazole-CDPK1, Pantoprazole-CDPK1, Betamethasone-CDPK1, and Bromocriptine-CDPK1complexes showed that due to the negative binding energy of all the complexes, these drugs have a strong binding to the CDPK1 protein and this binding is stronger for pantoprazole, Betamethasone, and Bromocriptine (Table 6).

**Table 4:** The Van der Waals, electrostatic, polar solvation, SASA, SAV, and binding energies of the DHFR-TRC-2533, DHFR-Ezetimibe, DHFR-Phenazopyridine, DHFR-Raloxifene, DHFR-Sulfasalazine, DHFR-Triamterene, DHFR- Zafirlukast complexes (kJ/mol), calculated by the MMPBSA method.

Energy	DHFR-TRC-2533	DHFR-Ezetimibe	DHFR-Phenazopyridine	DHFR-Raloxifene	DHFR-Sulfasalazine	DHFR-Triamterene	DHFR-Zafirlukast
Van der Waal energy (KJ/Mol)	− 0.068 ± 0.014	− 266.620 ± 54.209	− 73.507 ± 89.273	− 318.391 ± 80.20	− 365.160 ± 12.024	− 153.899 ± 100.447	− 368.545 ± 13.191
Electrostattic energy (KJ/Mol)	2.328 ± 0.526	− 20.809 ± 6.249	0.431 ± 0.751	− 4.763 ± 3.072	− 60.955 ± 7.529	− 2.952 ± 2.527	− 77.936 ± 11.707
Polar solvation energy (KJ/Mol)	− 35.473 ± 16.047	86.215 ± 12.999	46.840 ± 42.732	74.277 ± 21.86	181.786 ± 10.684	28.786 ± 17.774	205.886 ± 17.433
SASA energy (KJ/Mol)	0.021 ± 3.044	− 20.340 ± 4.342	− 6.090 ± 7.698	− 23.768 ± 6.02	− 21.941 ± 0.863	− 10.666 ± 7.014	− 29.918 ± 1.085
SAV energy (KJ/Mol)	0.000 ± 0.000	0.000 ± 0.000	0.000 ± 0.000	0.000 ± 0.000	0.000 ± 0.000	0.000 ± 0.000	0.000 ± 0.000
Binding energy (KJ/Mol)	− 33.192 ± 16.517	− 221.554 ± 55.106	− 32.326 ± 114.33	− 272.645 ± 69.17	− 266.270 ± 12.67	− 138.731 ± 94.791	− 270.513 ± 16.938

**Table 5:** The Van der Waals, electrostatic, polar solvation, SASA, SAV, and binding energies of the Cromolyn-PRS, Ergotamine-PRS, Montelukast-PRS, Cefexim-PRS, Lactulose-PRS complexes (kJ/mol), calculated by the MMPBSA method.

Energy	PRS-Halofuginone	PRS-Cromolyn	PRS-Ergotamine	PRS-Montelukast	PRS-Cefexim	PRS-Lactulose
Van der Waal energy (KJ/Mol)	− 147.319 ± 12.87	− 302.387 ± 39.421	− 0.002 ± 0.000	− 1.385 ± 9.761	− 219.407 ± 83.384	− 169.217 ± 77.693
Electrostattic energy (KJ/Mol)	− 7.490 ± 8.360	− 25.298 ± 9.663	0.093 ± 0.037	− 0.213 ± 1.023	− 14.203 ± 8.205	− 6.235 ± 5.086
Polar solvation energy (KJ/Mol)	36.095 ± 17.468	130.675 ± 25.661	− 89.933 ± 67.253	− 12.590 ± 23.867	68.374 ± 33.364	60.632 ± 27.290
SASA energy (KJ/Mol)	− 13.921 ± 1.036	− 21.702 ± 2.970	0.081 ± 3.715	0.396 ± 3.735	− 17.469 ± 6.351	− 13.970 ± 6.010
SAV energy (KJ/Mol)	0.000 ± 0.000	0.000 ± 0.000	0.000 ± 0.000	0.000 ± 0.000	0.000 ± 0.000	0.000 ± 0.000
Binding energy (KJ/Mol)	− 132.635 ± 16.980	− 218.711 ± 30.941	− 89.761 ± 67.048	− 13.793 ± 27.141	− 182.706 ± 61.846	− 128.790 ± 62.183

**Table 6:** The Van der Waals, electrostatic, polar solvation, SASA, SAV, and binding energies of the Omeprazole-CDPK1, Pantoprazole-CDPK1, Betamethasone-CDPK1, Bromocriptine-CDPK1complexes (kJ/mol), calculated by the MMPBSA method.

Energy	3sx9 inhibitor	Betamethasone	Bromocriptine	Omeprazole	Pantoprazole
Van der Waal energy (KJ/Mol)	− 0.022 ± 0.001	− 0.148 ± 0.013	− 0.016 ± 0.001	− 29.928 ± 51.160	− 178.544 ± 89.481
Electrostattic energy (KJ/Mol)	1.225 ± 0.389	− 0.888 ± 0.322	− 0.091 ± 0.159	− 1.505 ± 1.740	− 14.827 ± 8.208
Polar solvation energy (KJ/Mol)	− 36.054 ± 22.262	− 32.207 ± 55.558	− 33.730 ± 16.59	32.232 ± 72.173	73.582 ± 11.086
SASA energy (KJ/Mol)	− 0.194 ± 2.654	− 0.084 ± 3.021	0.025 ± 2.069	− 7.151 ± 9.415	− 13.249 ± 6.572
SAV energy (KJ/Mol)	0.000 ± 0.000	0.000 ± 0.000	0.000 ± 0.000	0.000 ± 0.000	0.000 ± 0.000
Binding energy (KJ/Mol)	− 34.807 ± 22.130	− 33.326 ± 55.474	− 33.812 ± 17.04	− 6.351 ± 87.611	− 133.037 ± 110.041

## Discussion

*T. gondii* is a ubiquitous intracellular parasite that infects humans and warm-blooded animals^25^. The rapid spread of drug-resistant parasites has led scientists to identify and use vulnerable points in *T. gondii* parasites to discover alternative drugs and eradicate or control infections associated with this parasite^26,27^. Therefore, the discovering new antiparasitic compounds and new targets for treatment is essential^28^. The development of strategies to target the key vulnerabilities in eukaryotic parasites is essential to identify new therapies against these infections. To abandon the difficult and expensive ways of classical drug discovery, targeted drug discovery on common and conserved pathogenic targets is increasing^29^. Considering the high costs and time-consuming nature of new drug discovery and development, reuse of old drugs for the treatment of common and rare diseases is increasingly becoming an attractive proposition, due to the use of compounds with lower side effects and overall costs and shorter drug development timelines^30^. Various data-driven and experimental approaches have been suggested to identify of repurposable drug candidates. The regulatory and phase III costs may remain more or less the same for a repurposed drug as for a new drug in the same indication, but there could still be substantial savings in preclinical and phase I and II costs^30,31^. Together, these advantages with lower average associated costs once failures can result in a less risky and more rapid return on investment in the development of repurposed drugs. In this study, to find an alternative treatment instead of common treatments against *T. gondii* infection, we examined the interaction of 2100 approved drugs with three TgDHFR, PRS, and CDPK1 proteins. Previous studies have shown that these proteins are suitable targets for the vulnerability and elimination of *T. gondii* parasite^18,19,23^. The results of our computational studies using molecular docking showed drugs with an inhibitory effects against each protein. Then, we performed molecular dynamics and MMPBSA analyses for more accurate evaluations. A competent inhibitor of the TgDHFR protein should have sufficient selectivity to inhibit parasiticidal activity without simultaneously inhibiting human DHFR (hDHFR). Selective drugs for TgDHFR protein should have a higher affinity for TgDHFR than hDHFR^32^. Since we chose 5-(4-(3-(2-methoxypyrimidin-5-yl) phenyl) piperazin-1-yl) pyrimidine-2, 4-diamine- TgDHFR complex as control. In this complex, the compound 5-(4-(3-(2-methoxypyrimidin-5-yl) phenyl) piperazin-1-yl) pyrimidine-2, 4-diamine binds to the TgDHFR with a higher affinity than the hDHFR one^32^. As a result, we chose drugs that have stronger interaction with TgDHFR compared to the 5-(4-(3-(2-methoxypyrimidin-5-yl) phenyl) piperazin-1-yl) pyrimidine-2, 4-diamine compound. Finally, Ezetimibe, Raloxifene, Sulfasalazine, Triamterene, and Zafirlukast drugs showed the best results against TgDHFR protein. Several studies have shown the remarkable ability of halofuginone (HF) to inhibit the activity of the prolyl-tRNA synthetase enzyme of *Plasmodium falciparum* and *T. gondii*^18,33,34^. This compound is a derivative of febrifugine, a natural quinazolinone alkaloid obtained from *Dichroa febrifuga* herb^18^. Considering the ability of this compound to inhibit the parasite, we decided to consider the Halofuginone-PRS complex as a control to choose the best alternative drugs. Cromolyn, Cefexim, and Lactulose drugs showed the strongest interactions with the PRS protein. The Toxoplasma life cycle is regulated by a family of CDPKs with no direct homologues in the human host^35^. Therefore, this protein can be a suitable target for drug design. C3-substituted pyrazolopyrimidine compounds are potent and selective inhibitors of CDPK1^24^. For this reason, we chose TgCDPK1 in a complex with a bumped kinase inhibitor, RM-1–132, as a control for drug selection against *T. gondii.* Finally, Pentaprazole, Betamethasone, and Bromocriptine drugs had the highest potential for interacting and inhibiting of the CDPK1 protein. Our computational studies showed that drugs mentioned above showed strong inhibitory properties against *T. gondii* DHFR, PRS, and CDPK1 proteins.

## Conclusion

In this study, we evaluated potential TgDHFR, TgPRS, and TgCDPK1 protein inhibitors against *T. gondii* using the computational screening of FDA-approved drugs. We performed a virtual screening of available FDA-approved drugs, and analysis via MD simulation, and MMPBSA. Finally, Ezetimibe, Raloxifene, Sulfasalazine, Triamteren and Zafirlukast drugs against TgDHFR protein, Cromolyn, cefixime and lactulose drugs against TgPRS protein and Pentaprazole, Betamethasone and Bromocriptine showed the best results against TgDHFR protein. These drugs may be tested for their beneficial role in toxoplasmosis infection, primarily in vitro, in vivo, and clinical trials.

## Material and methods

### Protein preparation

The structure of proteins of Dihydrofolate Reductase (DHFR) with PDB ID: 6AOH, Prolyl-tRNA Synthetase (PRS) with PDB ID: 5XIQ, and Calcium-Dependent Protein Kinase 1 (CDPK1) with PDB ID: 3SX9 from *T. gondii* were retrieved from protein data bank (www.rcsb.org). Proteins were prepared for molecular docking. The ligands, ions, and all the water molecules were removed from the PDB files. Charges and missing hydrogen atoms were added to the DHFR, PRS, and CDPK1 proteins in the AutoDock Tools environment and were saved in pdbqt format to be used in the following steps.

### Ligand preparation

2100 FDA-approved drugs were extracted from the PubChem database. Inappropriate, organic polymers and inorganic compounds were removed manually. Then the structures of the ligands were prepared by AutoDock Tools 4.2. The SDF format was converted to PDB format using OpenBabel (version 2.4.1). Nonpolar hydrogen bonds were integrated, Gasteiger-Marsili charges were assigned, atoms were set with AutoDock atom types, and rotatable bonds were assigned and saved in pdbqt format using AutoDock Tools 4.2.

### Binding pocket selection and generation of grid box for docking studies

The binding site of the TgDHFR, TgPRS, and TgCDPK1 proteins interact with the TRC-2533, halofuginone, and RM-1–132 ligands , respectively was analyzed by LigPlot software^36^. We also used the study that investigated the crystal structure of these proteins and their active site to characterize the grid box more precisely^24,37,38^. Summing up these results was used to locate the binding pockets for virtual screening. After marking the critical amino acids, grid boxes of 34 × 29 × 25 Å (*x*, *y*, and *z*) for TgCDPK1 protein, 32 × 94 × 72 Å (*x*, *y*, and *z*) for TgDHFR protein and 3.5 × 1.4 × 17 Å (*x*, *y*, and *z*) for TgPRS protein with 1-Å grid spacing were selected by using AutoDock Tools 4.2.

### Structure-based and ligand-based virtual screening using molecular docking

For structure-based virtual screening and rigid docking of selected drugs, we decided to utilize Smina Autodock software^39^. The Smina function is based on a similar search algorithm. It represents the implementing of a different set of scoring functions that can be applied to both the ligand binding and ranking processes^39^. The docked complexes were visualized and analyzed using PyMol and Ligplot + software^36,40^.

The Pharmit software^41^ generated the TgDHFR, TgPRS, and TgCDPK1 proteins pharmacophore model based on the TgDHFR complexed with TRC-2533, TgPRS in complex with halofuginone, and TgCDPK1 in complex with a bumped kinase inhibitor, RM-1–132. These models were applied for the virtual screening of small-molecule compounds that block the binding site of *T. gondii* DHFR, PRS, and CDPK1 proteins. Pharmit is a web server that searches small molecules based on their structural and chemical similarity to another small molecule with the aim of identifying molecules that bind to a target molecule^41^.The virtual screening was performed in FDA-approved libraries containing over FDA-approved 2100 drugs.

These molecules were screened by molecular docking calculations to evaluate their binding affinity to *T. gondii* DHFR, PRS, and CDPK1 to identify the molecules that could have the most promising results in in vitro calculations. Among the drugs with the strongest interaction (Lowest binding energy) with *T. gondii* DHFR, PRS, and CDPK1 proteins, we chose drugs with lower side effects and cost-effectiveness.

### Molecular dynamics simulation

The protein–ligand complexes with the strongest interactions among docked complexes were selected for further evaluation by MD simulations by the GROMACS 2018 package^42^ and OPLS-AA force field^43^. Docked complexes were selected and placed in a triclinic box with a distance of 1 nm from all edges^44^. The topology parameters for the protein structure were prepared by the GROMACS program. Topology preparation and configuration parameters of each ligand were used using the PRODRG server. After that, drug–protein complexes were immersed in a simulation box filled with SPC (Simple Point Charge) water molecules^45^. To neutralize the simulation box in terms of electrical charge, an appropriate number of ions were added to the environment of the box. Finally, Na and Cl ions were used instead of solvent molecules in the simulation box, and the medium was neutralized. Three-dimensional periodic boundary conditions were applied to the system. For all MD simulations, the energy minimization process was comprised of two parts: firstly, systems were balanced at 300 K for 100 ps using NVT (constant Number of particles, Volume, and Temperature) and secondly, a 1000 ps NPT (constant Number of particles, Pressure, and Temperature) equilibration of the system were performed using Parrinello–Rahman barostat to obtain constant temperature and pressure in 300 K and 1.0 bar. The long-range electrostatics was treated with Particle Mesh Ewald (PME) algorithm by 10 Å cutoff distance. Van der Waals (VDW) interactions were computed with a 1 nm cutoff. The Linear constraint (LINCS) algorithm was used to constrain the length of covalent bonds. Finally, for all MD simulations, the first 10 ns was taken as equilibrium time, leading to 40 ns with 2 fs time steps. After the necessary equilibrations, 100 ns production runs were performed for the docked complexes. To evaluate the stability of the protein–ligand complexes, RMSD, RMSF, RG, and SASA were calculated^46^.

### MMPBSA binding-free energy

Analysis of the docked complexes binding-free energies were performed by applying the single-trajectory MMPBSA method^47^ via the g_mmpbsa package^48^. In this method, the binding-free energy is the result of the free energy of the complex minus the sum of the free energies of the ligand and the protein, as follows:

$${\mathbf{\Delta Gbind}} \, = \, {\mathbf{\Delta Gcomplex}}{-}\left( {{\mathbf{\Delta Gligand}} \, + \, {\mathbf{\Delta GProtein}}} \right).$$

## Data Availability

All the PDB files were obtained from the RCSB protein data bank (http://www.rcsb.org/). FDA-approved drugs were extracted from the PubChem database (https://pubchem.ncbi.nlm.nih.gov/). The binding site of all the complexes was analyzed by LigPlot software (https://www.ebi.ac.uk/thornton-srv/software/LigPlus/). The grid box of proteins was selected by using AutoDock Tools 4.2. Also, dockings were performed using AutoDock (https://autodock.scripps.edu/download-autodock4/). MD simulations by the GROMACS 2018 package were performed (https://manual.gromacs.org/documentation/2018/download.html). The Pharmit software was used to generate the proteins pharmacophore model (https://pharmit.csb.pitt.edu/). Analysis of the docked complexes' binding-free energies was performed by the MMPBSA method (https://rashmikumari.github.io/g_mmpbsa/). All data generated or analyzed during this study are included in this article.
